# Unusual case of pelvic hydatid cyst of broad ligament mimicking an ovarian tumour

**DOI:** 10.1099/jmmcr.0.005057

**Published:** 2016-08-30

**Authors:** Alaa Abdullah, Reema Alsafi, Jamshaid Iqbal, Vincent Rotimi

**Affiliations:** ^1^​ Microbiology Department, Mubarak Al Kabeer Hospital, Kuwait City, Kuwait; ^2^​ Department of Pathology, Maternity Hospital, Kuwait City, Kuwait; ^3^​ Department of Microbiology, Faculty of Medicine, Kuwait University, Kuwait City, Kuwait

**Keywords:** Hydatid cyst, abdominal mass, surgical excision, albendazole

## Abstract

**Introduction:**

The diagnosis of hydatid cyst in female genital tract is rare and difficult. A high degree of clinical suspicion is needed for pre-operative investigations to exclude hydatid cyst of female pelvis. The objective of this presentation is to highlight a pelvic hydatid cyst that presented as an ovarian tumour.

**Case presentation:**

A 22-year-old female, presented with constipation and haematuria with acute urinary retention. On examination, a mass measuring 15×13 cm was palpable in the left iliac region reaching up to the umbilicus. It was smooth, movable and non-tender and a provisional diagnosis of ovarian teratoma was made pre-operatively. At laparotomy, a cystic mass was found attached to the broad ligament, excised, and a frozen section was sent for histopathology. Gross features were consistent with hydatid cyst; the cystic wall was white and there were multiple small thin-wall daughter cysts. Microscopic diagnosis with paraffin sections showed cystic lesions with laminated wall and scolices in the daughter cyst. Indirect haemagglutination test for specific antibodies was positive (128 IU). The patient responded well to surgical excision followed by albendazole administration.

**Conclusion:**

This case highlights the fact that a pelvic hydatid disease may resemble neoplastic ovarian cyst, clinically and radiologically. The possibility of pelvic hydatid disease should be included, in endemic areas where differential diagnosis of cystic ovarian lesions is needed, so that the patient can be managed accordingly.

## Introduction

Hydatid disease is a parasitic infection caused by the larval stage of cestode (tapeworm) *Echinococcus granulosus *and *Echinococcus multilocularis*. The disease is endemic in sheep and cattle grazing countries like India, Australia, Middle East, Africa and South America (Pawlowski *et al.*, 2001; Craig, 2003). It is transmitted by the ingestion of eggs. It most commonly affects the liver and lungs. The pelvic organs in females are rarely the primary site of cyst formation (Mandell *et al.*, 2014). Bickers (1970), after reviewing 532 cases of hydatid disease from an endemic area over a 20-year period, recorded 12 instances where hydatid cysts were present in the pelvis, only 2 of which were in the broad ligament. The aim of this report is to highlight a rare presentation of primary pelvic hydatid disease located in the broad ligament.

## Case report

A 22-year-old Ethiopian lady was admitted to Mubarak Al-Kabeer Hospital complaining of urinary retention and haematuria. There was no associated fever or loss of appetite. The patient also had constipation with dull abdominal pain for the past 2 weeks. Her menstrual cycle was regular and her last menstrual period was 16 days back. The patient is single and has been working in Kuwait as a house maid for 2 years. There was no history of recent travel. There was no history of tuberculosis nor were there dogs or any pets where the patient lived. The patient denied having been on a farm.

On general physical examination, the patient was thin built, well nourished and pale. Temperature was 36.8 °C, blood pressure was 125/65 mm Hg and pulse was 72 beats min^−1^. The patient’s respiratory, cardiovascular and neurological systems were normal. The abdomen was soft and lax with mild diffuse tenderness but there was no rigidity. There was no hepatosplenomegaly and no ascites. A mass measuring 15×13 cm was palpable in the left iliac region reaching up to the umbilicus that had smooth surface, was movable and non-tender. There was no lymphadenopathy.

## Investigations

Routine haematological parameters revealed the following: white blood cell 33.2×109 l^−1^, eosinophil count 0×109 l^−1^, neutrophil count 28.9×109 l^−1^, Hb 99 g l^−1^ and platelet 394×109 l^−1^. Beta human chorionic gonadotrophin was negative. Biochemical parameters, including the liver and kidney function tests, were normal.

Ultrasonography of the abdomen was ordered and it revealed a large pelvic and lower abdominal multi-septated cystic mass measuring 17×12 cm that was thought to be most likely from the left ovary. There was also bilateral hydronephrosis and hydroureter most likely due to pressure changes of distal ureters from the mass lesion. The other abdominal organs were normal and there was no free fluid. In order to properly assess the mass, a magnetic resonance imaging (MRI) of pelvis was done. It revealed a large multi-loculated cystic mass, 11×13.3×14 cm, occupying the whole pelvis and extending into the left lower abdominal quadrant, which grossly compressed and deviated the uterus anteriorly and to the right of the pelvis. The mass had compressed the mid-portion of the rectum causing prominence of its proximal part in keeping with the patient’s history of constipation. The ovaries could not be seen properly, but there was no evidence of lymphadenopathy, free fluid or localized collection in the pelvis. The radiological report suggested a cystic mass that might be a neoplastic ovarian cyst, e.g. cystic teratoma.

Thereafter, the patient was promptly transferred to the maternity hospital for further gynaecological consultation and laparoscopic exploration. The patient underwent exploratory laparotomy through an intra-umbilical midline incision till the symphysis pubis. A cystic mass was found occupying the left broad ligament and displacing the uterus to the right with the left fallopian tube stretched over the mass. Both ovaries were normal. Only partial removal of the cyst was possible as a small part of the cyst wall was adherent to the rectum and uterus. It was not possible to dissect the cyst wall completely and so drooling and marsupialisation was performed on the remaining cyst wall to avoid injury to the rectum and uterus. No other intra-abdominal pathology was found. The liver was explored and no lesions were found. Peritoneal toilet and irrigation with hypertonic saline of the cavity was done many times and a suction drain was left in the Douglas pouch. The mass was a cyst filled with clear fluid and multiple daughter cysts. It was sent for histopathology and serology and fluid aspirated from left broad ligament cyst was sent for culture.

## Diagnosis

**Fig. 1.**
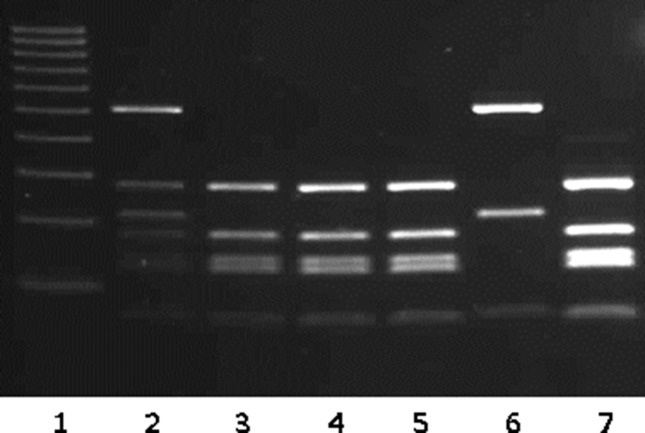
(a) Gross features of the hydatid cyst wall and the daughter cysts of variable size. (b) Microscopic paraffin sections of daughter cysts showing the laminated cyst wall. (c) Microscopic paraffin sections showing scolices in the daughter cyst.

Histopathology report of the frozen section was as follows: macroscopic diagnosis (Fig. 1a) revealed a left broad ligament cystectomy. The gross features were consistent with hydatid cyst. The cystic wall was white and measured 12×10×0.5 cm. There were multiple small thin-walled daughter cysts. Microscopic diagnosis with paraffin sections is shown in Fig. 1(b, c). There were cystic lesions with laminated wall and the scolices were noted in the daughter cyst as demonstrated in Fig. 1c.

Serology test was performed on the patient’s serum using indirect haemagglutination assay (IHA) for the quantitative detection of *E. granulosus* antibodies as an adjunct for the diagnosis of hydatid cysts. It was positive with a reading of 128 IU (diagnostic range ≥128 IU). Bacteriological culture of the fluid revealed no growth after 48 h. Blood sent for malarial parasite was also negative.

## Treatment

Surgical removal of the cyst was carried out during the exploratory laparotomy. After the diagnosis of pelvic hydatid disease was confirmed, the patient was managed with albendazole 400 mg twice a day orally for 28 days, followed by a period of rest and a repeat cycle again thereafter.

## Outcome and follow-up

The infectious disease hospital was informed of the case. The post-operative period was uneventful. The patient was stable and well. The drain was removed after 24 h. The liver function and renal function test were monitored twice weekly together with the complete blood count and coagulation profile. The patient was discharged on the 7th post-operative day to be followed up in the outpatient clinic.

## Discussion

Human echinococcosis (hydatidosis or hydatid disease) is caused by *E. granulosus* that causes cystic echinococcosis (CE) and *E. multilocularis* that is the causative agent of alveolar echinococcosis (AE). CE and AE are the two forms most frequently encountered. Our patient presented with CE.

Hydatid disease is usually acquired in childhood (Mandell *et al.*, 2014) The symptoms present several years after exposure and it may take 5–20 years before a diagnosis is made, which was probably the case in this patient. Human is an accidental host in the life cycle of *E. granulosus *(Mandell *et al.*, 2014). She most likely got the infection by ingesting the ova either by consuming contaminated unwashed vegetables or as a result of close association with pet dogs but she could not recall any pet in her house when she was young. However, this should not be surprising as the ova are partially resistant to desiccation and remain viable for many weeks, allowing delayed transmission to individuals with no direct contact with vector animals. Once in the intestinal tract, the ova hatch to form oncospheres then encyst in host viscera, developing over time to form mature larval cysts (Mandell *et al.*, 2014).

Infection with *E. granulosus* is estimated to occur in up to 2–6 % of endemic populations (Fuller & Fuller, 1981). A hyperendemic focus of hydatid disease was found in southwestern Ethiopia. Two tribes, the Dassanetch and Nyangatom, in the lower Omo River Valley were found to have a particularly high prevalence of the disease (Fuller & Fuller, 1981). The factors felt to contribute to this high incidence were the use of nurse dogs to clean up children; the close, familiar relationships between dogs and human; and a cluster village settlement pattern with its increased number of sheep–dog–man contacts (Fuller & Fuller, 1981). Since our patient is an Ethiopian, she is probably at high risk of being infected during her childhood in the endemic area and might not recall dogs in her childhood years.

The hydatid cyst tends to form in the liver in 50–70 % of cases, or in the lung 20–30 %, but may be found in any organ (Mandell *et al.*, 2014). Primary hydatid cyst in the pelvis as in this patient is rare and usually presents with pressure symptoms affecting the adjacent abdominal organs as was the case in our patient with pressure on the rectum leading to constipation and bladder with consequent urinary retention. For most cases, symptoms are often absent, and in many cases, infections are detected only incidentally by imaging studies. When symptoms do occur, they are usually due to the space-occupying effect of the enlarging cyst. In our patient, the cyst obstructed the ureters causing bilateral hydronephrosis. Of the 51 cases of hydatid disease reported in Kuwait between 1956 and 1960, only one was located in the pelvis (El Gazzar & McCreadie, 1962). In that study, the majority of patients were immigrants from other countries such as Iraq, Iran, Saudi Arabia and Jordan; only 5 (2.6 %) patients were Kuwaitis (El Gazzar & McCreadie, 1962). The mode of transmission of infection to pelvic area is not clear. The genital organs are considered to be the most affected areas in the pelvis in females. This can be attributed to the fact that the genital organs are relatively highly vascularized. Other reasons could be due to invasion from the connective tissue of the peritoneum of Douglas and suspensory ligaments (Terek *et al.*, 2000). Dissemination via lymphatics has been implicated as a possible route in primary pelvic hydatid disease (Luliano *et al.*, 2000).

It is very important that a correct pre-operative diagnosis is made since all precautions must be taken to prevent dissemination and seeding of the surgical field. Unfortunately, the presentation in this case was atypical and, as such, the recommendation of image-based stage-specific approach for CE by Brunetti *et al.* for the World Health Organization Informal Working Group on Echinococcosis (WHO-IWGE) (Brunetti *et al*., 2010) was not followed. Deaths have been reported due to anaphylactic shock resulting from spillage during excision or biopsy after a mistaken diagnosis of a retroperitoneal tumour. Infection that is suspected based on imaging studies may be confirmed by a specific enzyme-linked immunosorbent assay and western blot serology (Terek *et al.*, 2000). Serology is 80–100 % sensitive and 88–96 % specific for liver cyst but less sensitive for lung (50–56 %) or other organs (25–56 %) involvement. In this case, the IHA was positive. Eosinophilia is not a consistent or reliable finding. Imaging remains more sensitive with ultrasound scan (USS) and higher with computerized tomography (CT) and MRI than serodiagnostic techniques (Mandell *et al.*, 2014). USS and CT scan may demonstrate features like multi-locular appearance, a fluid level from hydatid sand and ultrasonic ‘water lily sign’. In this patient, abdominal USS and MRI gave the impression that the mass was a neoplastic ovarian cyst. Fine-needle aspiration cytology (FNAC) may help in establishing the diagnosis of uni-locular pelvic cystic mass but care must be taken to avoid incidence of anaphylactic reactions. FNAC may also show hooklets, scolices and laminated cyst wall. However, FNAC was not done in this case because neoplastic ovarian cyst was the provisional diagnosis.

According to WHO-IWGE report (Brunetti *et al*., 2010), there is no ‘best’ treatment for CE as no clinical trial has compared all the different modalities. The optimal treatment of symptomatic CE is total surgical resection. However, consensus by the WHO-IWGE experts is that adequate therapy should be based on image-based staging. Traditionally, because of risk of spreading infection due to cyst rupture, the recommended approach has been to visualize the cyst, remove a fraction of the fluid and instill a cysticidal agent such as hypertonic saline, cetrimide or 70–95 % ethanol to kill the germinal layer and daughter cysts before resection (El Gazzar & McCreadie, 1962) when the cyst is totally removed after 30 min of instillation. In our case, the cyst could not be removed completely, as a small part of the cyst wall was adherent to the rectum and uterus. The cyst wall was marsupialized followed by peritoneal toilet and irrigation of the abdominal cavity with hypertonic saline and a drain was left in the Douglas pouch. Laparoscopic surgery for cyst removal has been done in the past in less advanced cases in which spillage of contents is less likely to occur (Luliano *et al.*, 2000). Pre-operative treatment with albendazole for 1–3 months has been shown to significantly reduce the number of viable cysts found during surgery. Medical therapy for inoperative cysts with albendazole or mebendazole has provided improvement in most patients (55–79 %) but total cure in a smaller number (29 %). The preferred agent is albendazole because of its greater absorption from the gastrointestinal tract and higher plasma levels. It is given for 3 or more cycles at a dose of 400 mg twice a day for 4 weeks. However, for those less than 60 kg, 15 mg kg^−1^ day in 2 divided doses should be given, followed by 2 weeks of rest without therapy. The alternative agent, mebendazole, is poorly absorbed and must be taken at higher doses of 50–70 mg kg^−1^ day, for several months to achieve a therapeutic effect.

In conclusion, this case highlights the fact that a pelvic hydatid disease may resemble neoplastic ovarian cyst, clinically and radiologically. The possibility of pelvic hydatid disease should be included, in endemic areas where differential diagnosis of cystic ovarian lesions is needed, so that the patient can be managed accordingly.
